# Evaluation of immunophenotypic alterations of peripheral blood lymphocytes and their sub-sets in uncomplicated *P. Falciparum* infection

**DOI:** 10.1186/s12865-024-00638-8

**Published:** 2024-07-10

**Authors:** Samuel Antwi-Baffour, Benjamin Tetteh Mensah, Simon Aglona Ahiakonu, Dorinda Naa Okailey Armah, Samira Ali-Mustapha, Lawrence Annison

**Affiliations:** 1 Department of Medical Laboratory Sciences, School of Biomedical and Allied Health Sciences, College of Health Sciences, University of Ghana, Korle-Bu, P. O. Box KB 143, Accra, Ghana; 2 Department of Maternal and Child Health, School of Nursing, University of Ghana, Legon, Ghana; 3 Department of Medical Laboratory Technology, School of Medical Sciences, Accra Technical University, Accra, Ghana

**Keywords:** Haematological, Immunophenotypic, Parasite density, *P. Falciparum*, Lymphocytes and malaria

## Abstract

**Background:**

Malaria is a life-threatening parasitic disease typically transmitted through the bite of an infected Anopheles mosquito. There is ample evidence showing the potential of malaria infection to affect the counts of lymphocyte subpopulations in the peripheral blood, but the extent of alteration might not be consistent in all geographical locations, due to several local factors. Although Ghana is among the malaria-endemic countries, there is currently no available data on the level of alterations that occur in the counts of lymphocyte subpopulations during *P. falciparum* malaria infection among adults.

**Aim:**

The study was to determine the immunophenotypic alterations in the level of peripheral blood lymphocytes and their subsets in adults with uncomplicated P. *falciparum* malaria infection and apparently healthy participants.

**Methods:**

The study was a cross-sectional comparative study conducted in two municipalities of the Volta region of Ghana. Blood samples were collected from study participants and taken through serology (*P. falciparum*/Pan Rapid Diagnostic Kits), microscopy (Thick and thin blood films) and Haematological (Flow cytometric and Full blood count) analysis.

**Results:**

A total of 414 participants, comprising 214 patients with malaria and 200 apparently healthy individuals (controls) were recruited into this study. Parasite density of the malaria patients ranged from 75/µL to 84,364/µL, with a mean of 3,520/µL. It was also observed that the total lymphocytes slightly decreased in the *P. falciparum*-infected individuals (Mean ± SD: 2.08 ± 4.93 × 10^9^/L) compared to the control group (Mean ± SD: 2.47 ± 0.80 × 10^9^/L). Again, there was a significant moderate positive correlation between parasite density and haematocrit levels (*r* = 0.321, *p* < 0.001). Apart from CD45 + T-cells, more people in the control group had normal values for the lymphocyte subsets measured compared to the malaria patients.

**Conclusions:**

From the results obtained, there was high parasite density among the malaria patients suggestive of high intensity of infection in the case group. The malaria patients again showed considerable haematological alterations in lymphocyte sub-sets and the parasite density appeared to be strongly associated with CD4 + T-cell reduction. Also, the parasite density significantly associated with decreasing haematocrit levels. This indicates that lymphocyte subset enumeration can be used to effectively support malaria diagnosis.

## Background

Malaria is a life-threatening parasitic disease typically transmitted through the bite of an infected Anopheles mosquito which carries the erythrocytic protozoan of the phylum Apicomplexa and genus *Plasmodium* [1]. Malaria in humans is caused by five main species of *Plasmodium*, namely: *P. falciparum, P. vivax, P. ovale, P. malariae, and P. knowlesi* [1]. Among these species, *P. falciparum* and *P. vivax* pose the greatest threat to humans, but *P. falciparum* is considered the deadliest [2, 3]. The World Health Organization (WHO) defines malaria as uncomplicated when symptoms are present, but there are no clinical or laboratory signs to indicate severity or vital organ dysfunction [4]. It is refreshing to note that the vast majority of malarial infections cause uncomplicated malaria, with only approximately 1–2% of these episodes becoming severe [5].

Malaria parasites have been identified to disrupt the normal profile of immune cells in the peripheral blood. For example, there have been reports on alterations in the proportions of total leukocytes, total lymphocytes, Natural Killer cells, αβ, and γδ T-cell, and B-cell counts during *P. falciparum* and *P. vivax* infections [6, 7]. Severe *P. falciparum* malaria is also associated with increased plasma levels of a wide range of cytokines and markers of T cell activation and endothelial inflammation [8, 9]. It is also associated with T cell lymphopenia and impaired ability of peripheral blood T cells to produce some cytokines in vitro [9].

Furthermore, it has been postulated that, CD8 + T cells and the cytokines IFN-γ and TNF protect against pre-erythrocytic *Plasmodium* within hepatocytes, whereas CD4 + T cells restrict the growth of Plasmodium parasites in erythrocytes through secretion of cytokines, activation of macrophages and general direction of humoral immunity [10, 11]. The contribution of regulatory T cells in malaria infection has also been demonstrated, suggesting that the balance between pro-and anti-inflammatory cytokines is needed to monitor changes related to malaria [12, 13]. Although Ghana is among malaria-endemic countries (in 2022, WHO estimated that there were an estimated 5.3 million malaria patients with 11,557 estimated deaths recorded in Ghana) there is currently no available data on the level of alterations that occur in lymphocyte subpopulations during *P. falciparum* malaria infection among adults. Thus, this study, it is believed will fill the gap and inform the implementation of special steps when dealing with malaria patients, such as enumeration of peripheral lymphocyte cells for diagnostic purposes and to evaluate the immune status of malaria patients.

## Results

### Demographic and social features of the study participants

The study comprised four hundred and fourteen (414) participants made up of 214 patients with malaria and 200 apparently healthy individuals as controls. Other demographic and social features of the study participants obtained include age, gender, years of stay in the region, employment status, educational background, closeness of residence to stagnant water, and the use of stagnant water. The socio-demographic characteristics of the study population are shown in Table 1.

**Table 1:** Demographic features of the study participants

Features	Malaria patients (*N* = 214)		Control (*N* = 200)	
Age statistics				
Mean ± SD	31.58 ± 12.27		36.6 ± 10.06	
Median (Min – Max)	30.5 (9–58)		35.5 (6–61)	
Median (IQR)	30.5 (22–40)		35.5 (30–45)	
**Temperature**	^**o**^ **C** ^**o**^ **C**			
Median (IQR)	39.5 (37.5–40.6)		36.5 (36–37.5)	
	**Number**	**%**	**Number**	**%**
**Gender**				
Male	90	42.1	119	59.5
Female	124	57.9	81	40.5
**Years of stay in the region**				
2–10	164	76.6	150	75
11–20	40	18.7	30	15
21–30	7	3.27	15	7.5
31–40	1	0.47	5	2.5
**Employment status**				
Employed	118	55.1	102	51
Unemployed	91	42.5	96	48
Retired	3	1.4	2	1
**Education**				
Basic	60	28.0	61	30.5
Secondary	94	43.9	56	28
Tertiary	46	21.5	74	37
Uneducated	12	5.6	9	4.5
**Closeness of residence to stagnant water**				
Yes	148	69.2	44	22
No	64	29.9	156	78
**Use of ITN**				
Yes	105	49.1	187	93.5
No	109	50.9	13	6.5

### Enumeration of parasite density of the malaria patients

**Fig. 1:**
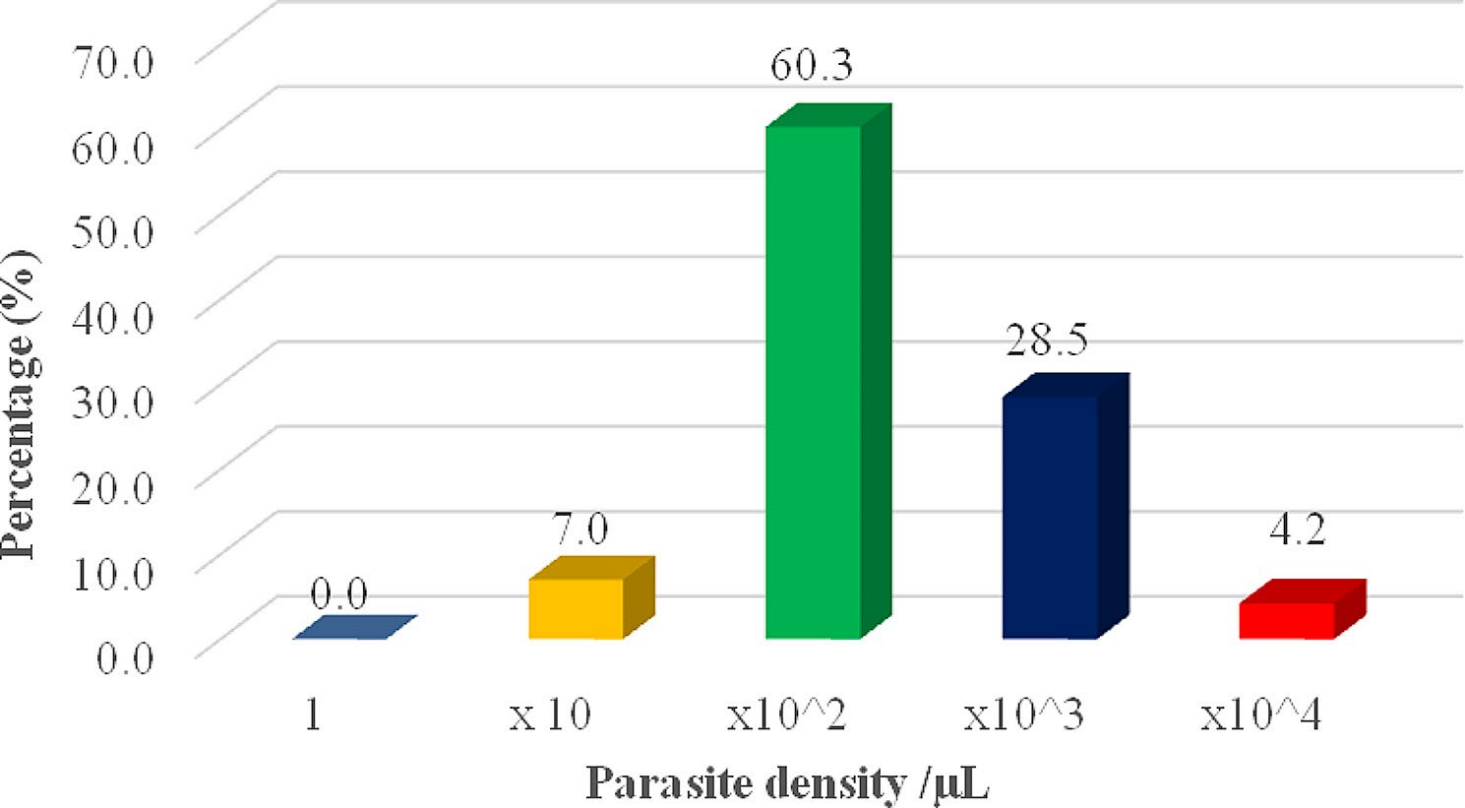
Parasite density of the *P. falciparum*-infected patients (malaria patients)

When the parasite density was analyzed among the malaria patients, they ranged from 75/µL to 84,364/µL, with a mean of 3,520/µL and most (129/214, 60.3%) had parasite density within x10^2^, whilst 28.5% had within x10^3^ (Fig. 1).

### General haematological parameters among the study participants

When comparing the various hematological parameters between malaria patients and the healthy control group, it was observed that the total lymphocyte counts were lower in the malaria patients (Mean ± SD: 2.08 ± 4.93 × 10^9^/L) compared to the control group (Mean ± SD: 2.47 ± 0.80 × 10^9^/L). Other notable decreases included haemoglobin (13.61 ± 1.22 g/dl vs. 11.53 ± 2.03 g/dl), haematocrit (1.27 ± 8.92 vs. 0.33 ± 0.06), mean corpuscular volume (170.00 ± 860.90 fL vs. 82.61 ± 9.03 fL), mean corpuscular haemoglobin (38.06 ± 48.21 pg vs. 28.18 ± 2.92 pg) and platelets (203.64 ± 78.74 × 109/L vs. 145.45 ± 70.04 × 109/L) (see Table 2).

**Table 2:** General haematological parameters among the study participants

Haematological Component	Mean ± SD *p*-value		
	Control group	Malaria patients group	
Red blood cells	4.50 ± 0.51 × 10^12^/L	4.07 ± 0.72 × 10^12^/L	0.0001*
Haemoglobin	13.61 ± 1.22 g/dl	11.53 ± 2.03 g/dl	0.0001*
White blood cells	6.43 ± 2.26 × 10^9^/L	6.64 ± 4.31 × 10^9^/L	0.5393
Neutrophils	3.36 ± 2.14 × 10^9^/L	4.20 ± 2.90 × 10^9^/L	0.000931*
Lymphocytes	2.47 ± 0.80 × 10^9^/L	2.08 ± 4.93 × 10^9^/L	0.2703
Monocytes	0.40 ± 0.18 × 10^9^/L	0.44 ± 0.24 × 10^9^/L	0.0571
Eosinophils	0.20 ± 0.19 × 10^9^/L	0.10 ± 0.21 × 10^9^/L	0.0001*
Basophils	0.06 ± 0.05 × 10^9^/L	0.05 ± 0.04 × 10^9^/L	0.0247*
Haematocrit	1.27 ± 8.92	0.33 ± 0.06	0.1247
Mean corpuscular volume	170.00 ± 860.90 fL	82.61 ± 9.03 fL	0.1380
Mean corpuscular haemoglobin	38.06 ± 48.21 pg	28.18 ± 2.92 pg	0.00293*
Mean corpuscular haemoglobin concentration	180.08 ± 164.97 g/dL	331.37 ± 58.37 g/dL	0.0001*
Red cell distribution-coefficient variation	2.09 ± 10.55	0.16 ± 0.05	0.00774*
Red cell distribution-standard deviation	50.74 ± 47.75 fL	49.41 ± 11.20 fL	0.6925
Platelets	203.64 ± 78.74 × 10^9^/L	145.45 ± 70.04 × 10^9^/L	0.0001*
Mean platelet volume	10.71 ± 1.02 fL	10.34 ± 1.13 fL	0.000537*
Platelet distribution width	16.05 ± 0.69	16.03 ± 0.57	0.7473
Plateletcrit	1.90 ± 0.61 mL/L	1.50 ± 0.72 mL/L	0.0002*
Platelet large cell count	57.01 ± 18.02 × 10^9^/L	43.74 ± 22.09 × 10^9^/L	0.0008*
Platelet large cell ratio	0.31 ± 0.07	0.31 ± 0.08	1.0

Mean parasite density of the malaria-infected group (malaria patients) = 3520.12 ± 11820.38069

*A *p*-value less than 0.05 is considered statistically significant

### Levels of lymphocyte subsets among the malaria patients

**Fig. 2:**
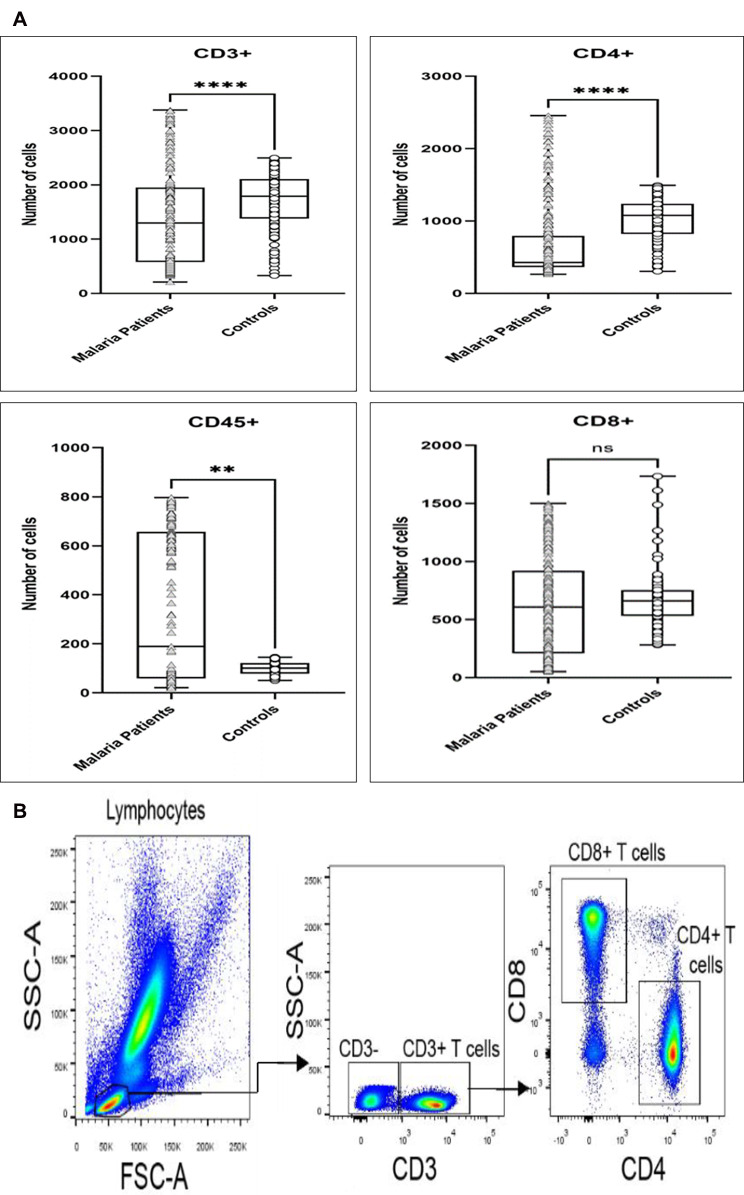
**A** shows the levels of CD3^+^, CD8^+^, CD4^+^, and CD 45^+^ cells in both the malaria patients and the control group whilst **B** shows the gating strategy used in acquiring the data. There was a significant difference in three of the subsets measured

Levels of lymphocyte subsets, CD3^+^, CD8^+^, CD4^+^, and CD45^+^ were measured among the two study groups based on Becton Dickinson (BD) reference rages [14]. Figure 2 A shows the difference in the level of lymphocyte subsets in each group based on the subset count. There were significant differences in the CD3^+^, CD4^+^, and CD45^+^ lymphocyte subsets between the malaria patients and the control group whilst CD8^+^ did not show significant difference.

### Independent samples t-test of the haematologic parameters of the participants

The independent samples t-test conducted revealed significant differences between the malaria patients and the control group, in terms of the means of several of their haematologic parameters. Similarly, results of the Z-ratio computed, highlighted significant differences between the malaria patients and control participants in terms of their lymphocytic parameters (Table 3).

**Table 3:** Z ratio results of the lymphocytic parameters of the study participants

	Control (*N* = 200) Number (%)	Malaria patients (*N* = 214) Number (%)	Z ratio	*p*-value
**CD3**				
Low	8 (4%)	80 (37.4%)	-8.297	< 0.0002
Normal	187 (93.5%)	97 (45.3%)	10.553	< 0.0002
High	5 (2.5%)	37 (17.3%)	-4.981	< 0.0002
**CD8**				
Low	0 (0%)	52 (24.3%)	-	-
Normal	193 (96.5%)	104 (48.6%)	10.817	< 0.0002
High	7 (3.5%)	58 (27.1%)	-6.597	< 0.0002
**CD4**				
Low	9 (4.5%)	136 (63.8%)	-	-
Normal	189 (94.5%)	51 (23.9%)	-12.986	< 0.0002
High	2 (1%)	26 (12.2%)	-	-
**CD45**				
Low	200 (100%)	104 (48.6%)	-	-
Normal	0	13 (6.1%)	-	-
High	0	97 (45.3%)	-	-

### Associations between parasite density and haematological parameters

There was a significant moderate positive correlation between parasite density and haematocrit levels (*r* = 0.321, *p =* 0.001). The coefficient of determination for this association was 0.103, indicating that parasite density and haematocrit levels share 10.3% of their variance. The other parameters were not significantly associated with parasite density (Table 4).

**Table 4:** Associations between parasite density and specific haematological parameters

Haematological parameters	*r*	*r* ^2^	*p*
White blood cell count	0.129	0.017	0.06
Neutrophil count	0.094	0.009	0.172
Lymphocyte count	0.089	0.008	0.194
Monocyte count	-0.013	0.000	0.852
Eosinophil count	0.006	0.000	0.932
Basophil count	0.045	0.002	0.509
Platelet count	0.088	0.001	0.198
Red blood cell count	-0.050	0.003	0.470
Haemoglobin level	-0.054	0.003	0.429
Haematocrit level	0.321**	0.103**	**0.001**

**Significant at 0.05 alpha level. From the table, it can be seen that only haematocrit level showed a significant correlation with parasite density among malaria patients

## Discussion

Although there are pieces of evidence showing the potential of malaria infection to affect the counts of lymphocytes and their subpopulations in the peripheral blood, the degree of effect might not be the same everywhere [9]. This is because, the pathogenesis as well as the disease outcome of malaria is associated with different factors such as the level of endemicity, host genetics, and parasite factors [15, 16]. So the ability of malaria parasites being able to perturb the normal profile of immune cells in the peripheral blood is well established [17] and this was evident in the current study. Subsequently, the main findings of the study showed that there was high intensity of malaria infection among the malaria patients, and they were characterized by considerable haematological alterations in lymphocyte subsets in particular CD4 + lymphocytes as well as a significant decrease in haematocrit levels. Although Ghana is a malaria-endemic country [18], not much has been done to elucidate the alterations that occur in the counts of lymphocytes and their subpopulations during P. *falciparum* malaria infection. However, this study has shown the possibility of alterations in lymphocyte subpopulations in the peripheral blood of P. *falciparum* malaria patients within the Volta Region of Ghana. Therefore, the study may inform the implementation of special steps needed to be taken when dealing with malaria patients, such as enumeration of peripheral lymphocyte sub-sets or populations to augment the other malaria diagnosis methods.

Regarding the employment status of the participants, the malaria patients had a higher percentage of employment with a few of them being retired. The trend was the same for the control group. Also, on the educational background, it was revealed that a greater number of the control group have attained basic education compared to the malaria patients but more of the malaria patients had secondary education compared to the control group. In terms of residence, the majority of the study group were known to have their houses close to stagnant water whilst the opposite was true for the control group. Again, with the use of insecticide-treated nets (ITNs), a greater number of the control group used ITNs than the malaria patients perhaps an indicator of their infection status.

In determining the degree of malaria infection (parasite density) among malaria patients, it was observed that their parasite density indicated a generally high parasite count. This finding was similar to a study by Awosolu et al., (2021) and Jalal et al., (2016) who found a relatively higher parasite density among their study participants [19, 20]. In high-transmission areas such as the current study location, it has been seen that a higher proportion of the population may be diagnosed with parasites based on microscopy findings but may be asymptomatic, and asymptomatic parasite densities often extend up to 10,000 parasites/µL [21]. Therefore, it is not surprising that microscopy employed in this study was able to show observation of the high intensity of the infection.

Regarding haematological parameters, it was observed that the malaria patients had a significantly lower red blood cell count, haemoglobin level, haematocrit level, mean corpuscular volume, mean corpuscular haemoglobin, mean corpuscular haemoglobin concentration, red cell distribution, platelet counts, mean platelet volume, platelet distribution width, plateletcrit, as well as platelet large cell count and ratio, than the control group. These findings concur with other studies that postulate that high parasitemias are known to exacerbate anaemia due to excessive haemolysis of parasitized red blood cells (RBCs) [22–25].

Furthermore, thrombocytopenia was also observed among the malaria patients which has also been noted by previous studies that increasing levels of *P*. *falciparum* parasite density results in a decreased platelet count via peripheral destruction [26]. Another study suggested immune-mediated destruction of circulating platelets as a possible cause of thrombocytopenia in malaria infections, especially those caused by *P*. *falciparum* [27]. Another observation in this study was that the malaria patients had a significantly higher neutrophil count than the control group. These findings parallel what was observed in the study of Kotepui et al., (2015) and is also consistent with the integral role that neutrophils play in the immune response to pathogens [28]. On another hand, the study recorded lower levels of eosinophils and basophils for the malaria patients than the control group in line with other studies [28].

Furthermore, a key objective of this study was to determine whether significant differences exist between the control group and the malaria patients about their levels of CD4^+^, CD3^+^, CD8^+^, and CD45^+^ lymphoid cells. This was to understand how malaria parasites affect the normal profile of immune cells in the peripheral blood [17]. From the study outcome, it was seen that more participants in the control group had normal CD3^+^, CD8^+,^ and CD4^+^ cells as compared with the malaria patients who showed depletion in the cells. Subsequently, the box and whisker plots showed significant differences in the CD3+, CD4+, and CD45 + lymphocyte subsets between the malaria patients and the control group whilst CD8 + did not show a significant difference (Fig. 2A). This finding was in line with earlier reports that T cell lymphopenia is a well-established feature of *P. falciparum* malaria with the depletion attributed to sequestration or apoptosis [29–32]. The CD45 + levels were, however, lower among the controls. This was because there have been suggestions that CD45 + cells have a functional role in hematopoietic cell activation and differentiation and therefore are seen to be expressed more in pathologic conditions rather than in healthy states [33]. Another study posited that human polymorphic variants that alter CD45 isoform expression are associated with autoimmune and infectious diseases, establishing CD45 as an important immunomodulator with a significant influence on disease burden [34, 35].

In determining the possible associations between the degree of malaria infection (parasite density) and specific leucocytic and haematological parameters among the malaria patients, it was seen that except for haematocrit levels, none of the parameters evaluated showed a significant association with parasite density. This is in line with the study of Bashawri et al., (2002) and Ekval, (2003), but in contrast to a previous study by McKenzie et al., (2005) which found a consistent positive relationship between leukocyte counts and parasite density in *Plasmodium*-infected patients [22, 23, 36].

By way of limitations, though the common cold and HIV were ruled out, other parasitic infections were not. It has also been proven that B cells and monocytes play a significant role in malaria-induced infection, however, the study did not enumerate CD 19 subsets of lymphocytes and CD14 due to limited resources and with the understanding that focusing on T cells will still help in achieving the study objective.

## Conclusions

The main conclusions of the study are that there was high parasite density among the malaria patients suggestive of high intensity of infection among them. It was also observed that *P. falciparum* malaria infection is characterized by considerable haematological alterations in lymphocyte sub-sets (CD3, CD4, and CD8) tested in the current study in particular and it appears to be associated with CD4 + lymphocytopenia and also significantly associated with decreasing haematocrit levels. This study has therefore shown that *P*. *falciparum* infections may alter lymphocyte subpopulation levels out of the normal range in malaria patients within the specific study area highlighting the need and usefulness of the enumeration of peripheral lymphocyte subsets to augment malaria diagnosis particularly when the CD4 + T-cells are low.

## Methods

### The aim, design, and setting of the study

The study aimed to determine immunophenotypic alterations in the level of peripheral blood lymphocytes and their subsets in adults with uncomplicated P. falciparum malaria infection and apparently healthy participants.

This was a cross-sectional comparative study comprising 414 participants (214 malaria patients and 200 controls) conducted from April 2018 to June 2019 in two municipalities of the Volta region of Ghana, namely: Ketu South Municipal District and Ave Dakpa, the capital of the Akatsi North District.

### The characteristics of participants

The study comprised malaria patients 18 years and above who consented to take part in the study. Healthy blood donors living in the study area were included as controls.

#### Inclusion criteria

All clinically confirmed adults (18 years and above) with uncomplicated P. *falciparum* malaria infection and who have stayed in the Volta region for more than one year.

#### Exclusion criteria

Patients not clinically confirmed with uncomplicated P. *falciparum* malaria infection and who have not stayed in the Volta region for more than one year. Also, patients with confirmed malaria infection but have recently been treated with anti-malaria drugs. Again, Patients with infections that can affect lymphocyte count (e.g. HIV, common cold) were also excluded.

### Sampling technique

A convenient sampling method was used to recruit the participants and they were selected based on their convenient accessibility. Five (5) milliliters of venous blood were collected by venipuncture from each participant into an EDTA tube. Whole blood samples and slide smears were sent for analyses daily at the Ketu South Municipal Hospital laboratory and the Fevers Unit Laboratory of the Korle-Bu Teaching Hospital. Clinical and demographic data were also recorded using the structured questionnaire.

### Laboratory investigations

Laboratory procedures employed included serological, microscopy, and haematological analysis.

### Serology

The Malaria Rapid Diagnostic Test kit was used for the initial screening of participants before the collection of venous blood samples. Finger prick blood samples were evaluated for the presence of malaria parasite antigen using a malaria RDT kit, First Response Malaria Ag Pf (Premier Medical Corporation Ltd, Gujarat, India), catalog number PI19FRC25s. The manufacturer’s procedure was followed to carry out the test. Briefly, the cassette was labeled with a patient identification (ID) number. The middle or ring finger of the patient was disinfected with 70% alcohol and allowed to air dry. The disinfected finger was pricked, and the first blood was wiped off with dry cotton. A capillary tube was held vertically to draw the whole blood specimen. The blood was transferred into the sample well marked (S) on the cassette and buffer was applied. Results were read within the time duration specified by the manufacturer. Following confirmation of a positive malaria test, 5 ml of blood was taken into an EDTA tube and used for the other tests.

### Microscopy

Thick and thin blood films stained with 3% Giemsa stain were examined microscopically. Briefly, for thick film, a drop of blood (about 6 µl in size) was placed on the center of a pre-labeled grease-free glass slide. Without delay, the blood was spread with a glass spreader held at a steep angle to achieve a thick smear in a circle the size of a dime (diameter 1–2 cm). This was allowed to air dry in a horizontal position. Then Giemsa stain (3% solution diluted with buffer) was applied to the thick film and allowed to stand for 30 min. After this time, the stain was washed off using phosphate buffer (pH = 7.3–7.4) and again allowed to air dry. The dried stained thick smear was finally viewed under a light microscope using the 100x oil immersion objective lens.

For the thin films, 2 µl of blood was placed on the Centre of a grease-free glass slide. The drop of blood was then spread with a glass spreader held at an angle of 45^o^ to obtain a thin film with a smooth tail end. This was air-dried in a horizontal position and then fixed with absolute methanol for two minutes. After the fixing, Leishman/Giemsa stain was applied on the thin film and allowed to stain for 30 min. The stain was then washed off using phosphate buffer (pH = 7.2–7.4) and also air dried. The stained thin film was then viewed under a light microscope (Olympus Optics Ltd. UK) using a 100x oil immersion objective lens for species identification.

#### Determination of parasitaemia

The prepared thick film was used for the estimation of parasitaemia. The number of parasites/µl of blood was determined by enumerating the number of parasites in relation to total WBC count from full blood count (FBC) and from the thin film using the formula below:

$$\:\frac{\text{N}\text{o}.\:\text{P}\text{a}\text{r}\text{a}\text{s}\text{i}\text{t}\text{e}\text{s}\:\times\:\:\text{W}\text{B}\text{C}\:\text{c}\text{o}\text{u}\text{n}\text{t}\:\text{f}\text{r}\text{o}\text{m}\:\text{F}\text{B}\text{C}}{\text{N}\text{o}.\:\text{o}\text{f}\:\text{W}\text{B}\text{C}\:\text{c}\text{o}\text{u}\text{n}\text{t}\text{e}\text{d}\:\text{i}\text{n}\:\text{t}\text{h}\text{e}\:\text{f}\text{i}\text{l}\text{m}.}=\text{N}\text{o}.\:\text{p}\text{a}\text{r}\text{a}\text{s}\text{i}\text{t}\text{e}\text{s}\:\text{p}\text{e}\text{r}\:{\upmu\:}\text{L}\:\text{o}\text{f}\:\text{b}\text{l}\text{o}\text{o}\text{d}$$

### Full blood count (FBC)

For each blood sample taken, a full blood count was done using a fully automated Haematology Analyzer following the manufacturer’s instructions (Mindrey BC-5150 Auto Haematology Analyzer). The blood samples were placed under the aspiration chamber of the Haematology Analyzer. The aspiration button was pressed for them to suck a portion of the sample into the analyzer for the parameters to be measured. Other parameter results were obtained via calculations.

### Flow cytometric analysis

To obtain absolute counts of lymphocytes, a dualplatform method (using a haematology instrument and a flow cytometer) was applied. A BD FACScalibur flow cytometer 4-color (Becton Dickinson, NJ, USA) was used for the identification and enumeration of lymphocyte subsets. Whole blood (50 µl) was incubated at room temperature with antibodies conjugated with the fluorochromes fluorescein isothiocyanate (FITC), phycoerythrin (PE), PE-Texas Red and PE-cyanine5 (PE-Cy5) to permit 4-colour analysis.

After the appropriate antibodies needed for surface staining were added to the whole blood and mixed thoroughly, the preparation was incubated for 30 min in the dark at room temperature. Afterwards, 2 mL of room temperature 1X eBioscience 1-step Fix/Lyse Solution (for RBC lyses) was added and then inverted gently. It was then incubated for 15–60 min at room temperature in the dark. The preparation was centrifuged at 500xg for 5 min at room temperature and the supernatant decanted. It was washed once with 2 mL Flow Cytometry Staining Buffer and spun again and the supernatant was decanted. The cell pellet was suspended in 200 µL Flow Cytometry Staining Buffer and analyzed. Before data acquisition, instrument parameters were checked and optimized using CaliBRITE beads (Becton Dickinson, NJ, USA). Data was acquired with BD CellQuest Pro software (Becton Dickinson) and analyzed with Paint-A-Gate software followed by MultiSET (both from Becton Dickinson, NJ, USA). As a control for appropriate lymphocyte gating, the mean percentages of CD4^+^ and CD8^+^ T cells were checked to ensure that they fall within a 10% range of the average percentage of CD3^+^ T-cells.

### Data analysis

Data collected were entered into Microsoft Excel 2010 (Microsoft Corp., USA), and imported into STATA statistical software version 12 (STATA Corp, TX, USA) for analysis. Descriptive statistics were used in summarizing the data obtained. The significance of the differences between the malaria-infected and control participants regarding the proportions of their CD3+, CD4+, CD8+, and CD45 + levels were assessed by calculating the Z ratio. Furthermore, an independent-sample t-test was conducted to compare the two study groups for differences in the means of their haematological parameters. The eta squared statistic was computed to determine the effect size and interpreted based on the guidelines of Cohen (1988) that 0.01 represents a small effect, 0.06 represents a moderate effect, and 0.14 represents a large effect.

With variables whose data violated the assumption of equal variances (which needed to be satisfied when using the independent sample t-test), values of the test statistic that compensate for the violation of this assumption were computed. Finally, the Pearson product-moment correlation was used to determine associations between parasite density and each of white blood cell count, platelet count, red blood cell count, haemoglobin, and haematocrit levels. These associations were presented using the Pearson product-moment correlation coefficient. *P*-values that were below 0.05 were considered statistically significant.

## Data Availability

The datasets used and/or analyzed during the current study are available from the corresponding author upon reasonable request.

## Abbreviations

BD: Becton Dickinson
CD: Cluster of differentiation
CI: Confidence Interval
EDTA: Ethylenediaminetetraacetic
FBC: Full Blood Count
FITC: Fluorochromes fluorescein isothiocyanate
HIV: Human immunodeficiency virus
ID: Identification number
IFN: Interferon
ITNs: Insecticide Treated nets
*P. falciparum*: *P. falciparum*
PE: Phycoerythrin
PE-Cy5: PE-cyanine5
RDT: Rapid diagnostic test
SD: Standard deviation
TNF: Tissue Necrosis Factor
WBC: White blood cells
WHO: World Health Organization

## References

[1] Sutherland CJ, Tanomsing N, Nolder D, Oguike M, Jennison C, Pukrittayakamee S (2010). Two nonrecombining sympatric forms of the human malaria parasite Plasmodium Ovale occur globally. J Infect Dis.

[2] Cox-Singh J, Hiu J, Lucas SB, Divis PC, Zulkarnaen M, Chandran P (2010). Severe malaria-a case of fatal Plasmodium knowlesi infection with post-mortem findings: a case report. Malar J.

[3] Greenwood BM, Fidock DA, Kyle DE, Kappe SHI, Alonso PL, Collins FH (2008). Malaria: progress, perils, and prospects for eradication. J Clin Invest.

[4] WHO. World Malaria Report 2015.

[5] Bartoloni A, Zammarchi L (2012). Clinical aspects of uncomplicated and severe malaria. Mediterr J Hematol Infect Dis.

[6] Lisse IM, Aaby P, Whittle H, Knudsen K (1994). A community study of T lymphocyte subsets and malaria parasitemia. Trans R Soc Trop Med Hyg.

[7] Worku S, Bjorkman A, Troye-Blomberg M, Jemaneh L, Farnert A, Christensson B. Lymphocyte activation and subset redistribution in the peripheral blood in acute malaria illness: distinct T cell patterns in P. Falciparum and P. Vivax infections. Clin Exp Immunol. 1997;108:34–41. 10.1046/j.1365-2249.1997.d01-981.x PMC1904634 9097908

[8] Krupka M, Seydel K, Feintuch CM, Yee K, Kim R, Lin CY (2012). Mild P. falciparum malaria following an episode of severe malaria is associated with induction of the interferon pathway in Malawian children. Infect Immun.

[9] Kemp K, Akanmori BD, Adabayeri V, Goka BQ, Kurtzhals JA, Behr C, Hviid L (2002). Cytokine production and apoptosis among T cells from patients under treatment for P. Falciparum malaria. Clin Exp Immunol.

[10] Depinay N, Franetich JF, Grüner AC, Mauduit M, Chavatte JM, Luty AJ (2011). Inhibitory effect of TNF-alpha on malaria pre-erythrocytic stage development: influence of host hepatocyte/parasite combinations. PLoS ONE.

[11] Imai T, Shen J, Chou B, Duan X, Tu L, Tetsutani K (2010). Involvement of CD8 + T cells in protective immunity against murine blood-stage infection with Plasmodium Yoelii 17XL strain. Eur J Immunol.

[12] Andrade BB, Reis-Filho A, Souza-Neto SM, Clarencio J, Camargo LM, Barral A (2010). Severe Plasmodium Vivax malaria exhibits marked inflammatory imbalance. Malar J.

[13] Bueno LL, Morais CG, Araujo FF, Gomes JA, Correa-Oliveira R, Soares IS (2010). Plasmodium Vivax: induction of CD4 + CD25 + FoxP3 + regulatory T cells during infection are directly associated with level of circulating parasites. PLoS ONE.

[14] http://www.bdbiosciences.com/ds/europe/tds/23-5351.pdf. Accessed: 01/06/2019.

[15] Trape JF, Rogier C, Konate L, Diagne N, Bouganali H, Canque B, Legros F, Badji A, Ndiaye G, Ndiaye P, Brahim K, Faye O, Druilhe P, Da Silva LP (1994). The Dielmo project: a longitudinal study of natural malaria infection and the mechanisms of protective immunity in a community living in a holoendemic area of Senegal. Am J Trop Med Hyg.

[16] Allen SJ, O’Donnell A, Alexander ND, Alpers MP, Peto TE, Clegg JB, Weatherall DJ (1997). Thalassemia protects children against disease caused by other infections as well as malaria. Proc Natl Acad Sci.

[17] Kassa D, Petros B, Mesele T, Hailu E, Wolday D (2006). Characterization of peripheral blood lymphocyte subsets in patients with acute P. falciparum and P. Vivax malaria infections at Wonji Sugar Estate, Ethiopia. Clin Vaccine Immunol.

[18] Hommerich L, von Oertzen C, Bedu-Addo G, Holmberg V, Acquah PA, Eggelte TA, Bienzle U, Mockenhaupt FP (2007). Decline of placental malaria in southern Ghana after the implementation of intermittent preventive treatment in pregnancy. Malar J.

[19] Awosolu OB, Yahaya ZS, Farah Haziqah MT, Prevalence (2021). Parasite density and determinants of Falciparum Malaria among Febrile Children in some Peri-urban communities in Southwestern Nigeria: a cross-sectional study. Infect Drug Resist.

[20] Jalal AB, Gasim IG, Amani HK, Leana ME, Ishag A (2016). Malaria Parasite Density Estimation using actual and assumed white blood cells count in children in Eastern Sudan. J Trop Pediatr.

[21] Imwong M, Stepniewska K, Tripura R, Peto TJ, Lwin KM, Vihokhern B, Wongsaen K, von Seidlein L, Dhorda M, Snounou G, Keereecharoen L, Singhasivanon P, Sirithiranont P, Chalk J, Nguon C, Day NP, Nosten F, Dondorp A, White NJ (2016). Numerical distributions of Parasite densities during asymptomatic Malaria. J Infect Dis.

[22] Ekvall H (2003). Malaria and anemia. Curr Opin Hematol.

[23] Bashawri LA, Mandil AA, Bahnassy AA (2002). Ahmed Malaria: hematological aspects Ann. Saudi Med.

[24] Suwanarusk R, Cooke BM, Dondorp AM, Silamut K, Sattabongkot J, White NJ, Udomsangpetch R (2004). The deformability of red blood cells parasitized by P. Falciparum and P. Vivax. J Infect Dis.

[25] Saravu K, Rishikesh K, Parikh CR (2014). Risk factors and outcomes stratified by severity of acute kidney injury in malaria. PLoS ONE.

[26] Shamez Ladhani O. Cole,3 Ken Kowuondo1 and Charles R. J. C. Newton. Changes in white blood cells and platelets in children with falciparum malaria: relationship to disease outcome. Br J Haematol. 2002;119:839–47. 10.1046/j.1365-2141.2002.03904.x 12437669

[27] Pain A, Ferguson DJP, Kai O (2001). Platelet-mediated clumping of P. falciparum erythrocytes is a common adhesive phenotype and is associated with severe malaria. Proc Natl Acad Sci USA.

[28] Kotepui M, Piwkham D, PhunPhuech B, Phiwklam N, Chupeerach C, Duangmano S (2015). Effects of malaria parasite density on blood cell parameters. PLoS ONE.

[29] Kemp HL, Kern KP. What is the cause of lymphopenia in malaria? Infect Immun. 2000;68(10):6087–9. 10.1128/iai.68.10.6087-6089.2000 PMC101581 11203040

[30] Kotepui M, Phunphuech B, Phiwklam N, Chupeerach C, Duangmano S (2014). Effect of malarial infection on haematological parameters in population near Thailand-Myanmar border. Malar J.

[31] Muwonge H, Kikomeko S, Sembajjwe LF, Seguya A, Namugwanya C (2013). How Reliable are hematological parameters in Predicting Uncomplicated P. Falciparum Malaria in an endemic region?. ISRN Trop Med.

[32] Desta M, Ayenew T, Sitotaw N (2018). Knowledge, practice, and associated factors of infection prevention among healthcare workers in Debre Markos referral hospital, Northwest Ethiopia. BMC Health Serv Res.

[33] Nakano A, Harada T, Morikawa S, Kato Y (1990). Expression of leukocyte common antigen (CD45) on various human leukemia/lymphoma cell lines. Acta Pathol Jpn.

[34] Elma Z, Tchilian, Peter CL, Beverley (2006). Altered CD45 expression and disease. Trends Immunol.

[35] Hermiston ML, Xu Z, Weiss A (2003). CD45: a critical regulator of signaling thresholds in immune cells. Annu Rev Immunol.

[36] McKenzie FE, Prudhomme WA, Magill AJ, Forney JR, Permpanich B, Lucas C, Gasser RA, Wongsrichanalai C (2005). White blood cell counts and malaria. J Infect Dis.

